# Supplementary material for: Genome-wide characterization of WRKY gene family in Helianthus annuus L. and their expression profiles under biotic and abiotic stresses
Source: PLoS One. 2020 Dec 3;15(12):e0241965. doi: 10.1371/journal.pone.0241965 (PMC7714227; doi:10.1371/journal.pone.0241965)
Supplement: S1 Table — (DOCX) [file pone.0241965.s001.docx]

S1 Table. Primers for qRT-PCR.

| Gene name | Forward | Reverse |
| --- | --- | --- |
| *ACT2* (reference gene) | GATTTGCCGGTGATGATGCT | TCCATGTCATCCCAGTTGCT |
| *HaWRKY3* | TTGCCGTGTGAAGAAGAGGG | GGAGAGTGATTGTGTCGACCTT |
| *HaWRKY5* | AGGCCCGAAAGGCTACAAAA | TTCCTTGGGTTTGGGCTGTT |
| *HaWRKY9* | CATACAAAGGGCAGCACACG | AAATGATGCCGGAGGGAAGG |
| *HaWRKY10* | CCTCACCTGCAGCTCATCAA | TGGATGGTTGTGTTGGCCTT |
| *HaWRKY13* | CATTAGCCGGAAGCACCAGA | CATCGCTGTCGTTGTCGTTC |
| *HaWRKY14* | GTGCGTACCAGCACAAAAGG | CTTGCTGGACAACCCCTCAT |
| *HaWRKY16* | GCGCAAATACGGGCAGAAAA | AGCCAAAAGGTTCGGGTTCA |
| *HaWRKY22* | TCGCCCGAAGCTAGAGACTA | TAACCGTCGTCAGCGTTACC |
| *HaWRKY29* | ATCACCCGAAACCGCAATCT | ACCGGACCTACTCCGATCAA |
| *HaWRKY30* | GCAGCGTTTCATTGGGTTGT | ACAACTACTCTCGGCTCCCT |
| *HaWRKY35* | TGGTGCTATGGCTTCACCTG | CACCAAGTCAACGGGCCTAT |
| *HaWRKY38* | ATCGGCGTAGCAATAGTGGG | ACACCTCGGTTTGTGTTGGT |
| *HaWRKY48* | CGTTTGCTAGTTGTCCAGCG | TACAGGATGCACCGCAAGTT |
| *HaWRKY52* | TTGATGGAGGTCCGGTTTGG | CATCCCCTTCGCCGTCATAA |
| *HaWRKY55* | ATCCGGTTCGGGTTTGGTTA | TCGGTTTCTCGATTGGGTGT |
| *HaWRKY57* | ATTCCTTAGCCCTGAACCCG | GTGGAGGCACAATATCCCCA |
| *HaWRKY59* | AGCGAGTGGAAAGGTCATCC | TCGAGGCGTCATTGGATACG |
| *HaWRKY74* | TGAGGCTACTGGTGCTTTGG | TTGGGTTCCAGCTTCCGTTT |
| *HaWRKY77* | AGATGTTGATTGCCGGTCGT | ATCCGTCCCCTCAACAACAC |
| *HaWRKY79* | TCAACCGCCGATGTTTCAGA | GTAGAACCGCCGCTACACTT |
| *HaWRKY81* | AGCTCAGGCCTAAACCGAAC | CGTCACAAGGGTTAGGCTGT |
| *HaWRKY82* | ATCCGAATTTCTCTGCCGCT | GTAGTTGCGCTGCCATTGTT |
| *HaWRKY89* | ACTCGGCTCCAATACCCTCT | CTGCTGAGTTGGACCGGAAT |
