# Supplementary material for: Genome-wide characterization of WRKY gene family in Helianthus annuus L. and their expression profiles under biotic and abiotic stresses
Source: PLoS One. 2020 Dec 3;15(12):e0241965. doi: 10.1371/journal.pone.0241965 (PMC7714227; doi:10.1371/journal.pone.0241965)
Supplement: S2 Table — (DOCX) [file pone.0241965.s002.docx]

S2 Table. Basic information regarding the presence of WRKY genes in sunflower.

| **Gene name** | **Gene ID** | **Chr** | **Genomic position** | | **Protein length (AA)** | **pI** | **Mw (kDa)** | **Strand** | **Group** |
| --- | --- | --- | --- | --- | --- | --- | --- | --- | --- |
|  |  |  | **Start** | **End** |  |  |  |  |  |
| *HaWRKY1* | Ha1_00044063 | Ha1 | 79910151 | 79912576 | 491 | 7.29 | 55.10 | minus | I |
| *HaWRKY2* | Ha1_00044349 | Ha1 | 97882925 | 97885011 | 275 | 9.92 | 31.06 | plus | IIc |
| *HaWRKY3* | Ha1_00044636 | Ha1 | 122318712 | 122322793 | 142 | 9.82 | 17.13 | minus | IIc |
| *HaWRKY4* | Ha3_00034844 | Ha3 | 44888934 | 44891981 | 293 | 10.11 | 32.63 | plus | I |
| *HaWRKY5* | Ha3_00035328 | Ha3 | 83537389 | 83537776 | 93 | 9.86 | 10.75 | plus | IIc |
| *HaWRKY6* | Ha3_00036250 | Ha3 | 143547469 | 143555327 | 135 | 9.00 | 15.52 | plus | I |
| *HaWRKY7* | Ha3_00036678 | Ha3 | 164224993 | 164226438 | 245 | 6.37 | 28.03 | minus | IIa |
| *HaWRKY8* | Ha3_00036976 | Ha3 | 177085591 | 177087707 | 308 | 5.46 | 34.79 | plus | IIIa |
| *HaWRKY9* | Ha3_00036977 | Ha3 | 177097957 | 177099130 | 325 | 5.32 | 36.47 | plus | IIIa |
| *HaWRKY10* | Ha4_00024227 | Ha4 | 37611807 | 37612771 | 268 | 5.82 | 30.46 | plus | IIc |
| *HaWRKY11* | Ha4_00024236 | Ha4 | 38156584 | 38159000 | 284 | 9.72 | 30.30 | minus | IId |
| *HaWRKY12* | Ha4_00024857 | Ha4 | 86933637 | 86934050 | 98 | 9.52 | 11.11 | plus | IIe |
| *HaWRKY13* | Ha4_00025692 | Ha4 | 164590074 | 164591047 | 182 | 5.16 | 19.71 | minus | IIe |
| *HaWRKY14* | Ha4_00025878 | Ha4 | 176614847 | 176616796 | 346 | 9.70 | 38.33 | minus | IId |
| *HaWRKY15* | Ha4_00026145 | Ha4 | 205997073 | 205999540 | 445 | 6.45 | 47.27 | plus | IIb |
| *HaWRKY16* | Ha5_00004294 | Ha5 | 2552227 | 2553152 | 237 | 8.35 | 25.92 | minus | IIb |
| *HaWRKY17* | Ha5_00004382 | Ha5 | 6117269 | 6118282 | 263 | 9.84 | 28.32 | minus | WKKY |
| *HaWRKY18* | Ha6_00047661 | Ha6 | 7634441 | 7636050 | 324 | 6.59 | 37.19 | minus | IIe |
| *HaWRKY19* | Ha6_00047844 | Ha6 | 14441295 | 14443510 | 267 | 6.14 | 30.24 | plus | IIc |
| *HaWRKY20* | Ha6_00048370 | Ha6 | 38834280 | 38835466 | 307 | 5.62 | 35.16 | plus | IIe |
| *HaWRKY21* | Ha6_00049148 | Ha6 | 79712170 | 79716297 | 347 | 9.95 | 38.99 | minus | I |
| *HaWRKY22* | Ha7_00045864 | Ha7 | 23919557 | 23920697 | 303 | 5.30 | 33.77 | minus | IIIa |
| *HaWRKY23* | Ha7_00045869 | Ha7 | 24265591 | 24266730 | 309 | 5.28 | 34.48 | plus | IIIa |
| *HaWRKY24* | Ha7_00046391 | Ha7 | 54156071 | 54159283 | 384 | 9.36 | 42.94 | minus | I |
| *HaWRKY25* | Ha7_00047314 | Ha7 | 101158887 | 101161518 | 324 | 6.05 | 36.23 | plus | IIIa |
| *HaWRKY26* | Ha7_00047446 | Ha7 | 108737256 | 108739887 | 324 | 6.05 | 36.23 | minus | IIIa |
| *HaWRKY27* | Ha8_00040991 | Ha8 | 14369591 | 14372927 | 464 | 7.07 | 50.78 | minus | I |
| *HaWRKY28* | Ha8_00041042 | Ha8 | 16954203 | 16960291 | 206 | 9.01 | 24.00 | minus | IIc |
| *HaWRKY29* | Ha8_00042335 | Ha8 | 125469544 | 125471147 | 390 | 6.57 | 43.19 | minus | I |
| *HaWRKY30* | Ha8_00042676 | Ha8 | 155503968 | 155507407 | 581 | 6.09 | 63.10 | minus | I |
| *HaWRKY31* | Ha8_00042907 | Ha8 | 180597105 | 180599286 | 441 | 9.00 | 48.03 | plus | IIb |
| *HaWRKY32* | Ha9_00011344 | Ha9 | 54325677 | 54332397 | 366 | 6.72 | 40.13 | minus | I |
| *HaWRKY33* | Ha9_00011438 | Ha9 | 61812802 | 61813910 | 253 | 9.88 | 27.61 | minus | WKKY |
| *HaWRKY34* | Ha9_00011781 | Ha9 | 96258562 | 96260485 | 206 | 5.20 | 23.30 | minus | IIc |
| *HaWRKY35* | Ha9_00013049 | Ha9 | 200266061 | 200268365 | 490 | 6.51 | 53.76 | plus | I |
| *HaWRKY36* | Ha9_00013316 | Ha9 | 214757544 | 214758398 | 253 | 10.43 | 28.99 | minus | IId |
| *HaWRKY37* | Ha9_00013796 | Ha9 | 241559196 | 241561365 | 229 | 8.43 | 25.97 | minus | IIIa |
| *HaWRKY38* | Ha10_00000492 | Ha10 | 25872888 | 25875224 | 347 | 9.58 | 38.78 | plus | IId |
| *HaWRKY39* | Ha10_00000902 | Ha10 | 52904543 | 52905906 | 221 | 5.50 | 25.66 | minus | IId |
| *HaWRKY40* | Ha10_00001096 | Ha10 | 65302668 | 65303929 | 315 | 6.46 | 35.72 | plus | IIIa |
| *HaWRKY41* | Ha10_00001107 | Ha10 | 66473378 | 66474753 | 325 | 8.33 | 37.29 | plus | IIIa |
| *HaWRKY42* | Ha10_00001194 | Ha10 | 71885525 | 71886840 | 321 | 6.56 | 36.72 | plus | IIIa |
| *HaWRKY43* | Ha10_00002013 | Ha10 | 130442899 | 130444396 | 307 | 6.27 | 34.63 | minus | IIIa |
| *HaWRKY44* | Ha10_00002386 | Ha10 | 165111355 | 165113856 | 211 | 5.97 | 22.98 | minus | IIe |
| *HaWRKY45* | Ha10_00002727 | Ha10 | 192838907 | 192842993 | 309 | 8.53 | 33.42 | plus | IIb |
| *HaWRKY46* | Ha10_00002822 | Ha10 | 200203096 | 200210111 | 290 | 9.69 | 31.53 | minus | IId |
| *HaWRKY47* | Ha10_00003216 | Ha10 | 237266513 | 237268440 | 475 | 6.58 | 54.28 | plus | I |
| *HaWRKY48* | Ha10_00003344 | Ha10 | 247804267 | 247806255 | 435 | 6.41 | 47.87 | minus | I |
| *HaWRKY49* | Ha10_00003426 | Ha10 | 252405298 | 252413742 | 483 | 5.36 | 52.71 | plus | IIb |
| *HaWRKY50* | Ha10_00003662 | Ha10 | 269803747 | 269804683 | 237 | 10.34 | 25.65 | minus | WKKY |
| *HaWRKY51* | Ha11_00028458 | Ha11 | 1582793 | 1583560 | 88 | 9.74 | 10.48 | minus | WKKY |
| *HaWRKY52* | Ha11_00030175 | Ha11 | 119212228 | 119216654 | 679 | 6.05 | 74.25 | minus | I |
| *HaWRKY53* | Ha12_00031802 | Ha12 | 24482403 | 24483662 | 255 | 10.44 | 27.94 | minus | WKKY |
| *HaWRKY54* | Ha12_00031805 | Ha12 | 24574664 | 24575598 | 103 | 9.92 | 11.82 | plus | WKKY |
| *HaWRKY55* | Ha12_00032116 | Ha12 | 41677521 | 41678818 | 183 | 7.04 | 20.78 | minus | IIc |
| *HaWRKY56* | Ha12_00033229 | Ha12 | 110971394 | 110973617 | 418 | 7.68 | 46.03 | minus | IIb |
| *HaWRKY57* | Ha12_00033722 | Ha12 | 158293177 | 158295039 | 162 | 9.45 | 18.42 | plus | IIc |
| *HaWRKY58* | Ha13_00014186 | Ha13 | 8104116 | 8104576 | 109 | 6.07 | 12.37 | plus | I |
| *HaWRKY59* | Ha13_00014680 | Ha13 | 60596959 | 60599004 | 277 | 8.56 | 31.20 | minus | IIc |
| *HaWRKY60* | Ha13_00014812 | Ha13 | 76595883 | 76596879 | 225 | 9.55 | 25.09 | plus | WKKY |
| *HaWRKY61* | Ha13_00015909 | Ha13 | 158739856 | 158741280 | 145 | 6.91 | 16.41 | minus | I |
| *HaWRKY62* | Ha14_00018327 | Ha14 | 113522433 | 113523706 | 169 | 9.30 | 19.02 | minus | IIc |
| *HaWRKY63* | Ha14_00019068 | Ha14 | 158188537 | 158189717 | 138 | 9.54 | 15.83 | minus | IIc |
| *HaWRKY64* | Ha14_00019831 | Ha14 | 198438866 | 198443661 | 283 | 5.62 | 30.96 | plus | IIe |
| *HaWRKY65* | Ha14_00020365 | Ha14 | 223460736 | 223462949 | 290 | 6.04 | 32.48 | minus | IIIa |
| *HaWRKY66* | Ha14_00020367 | Ha14 | 223577850 | 223579359 | 215 | 9.42 | 23.89 | minus | IIIa |
| *HaWRKY67* | Ha14_00020369 | Ha14 | 223672945 | 223674223 | 250 | 8.00 | 27.99 | minus | IIIa |
| *HaWRKY68* | Ha15_00037623 | Ha15 | 1907645 | 1909000 | 316 | 8.60 | 36.42 | plus | IIIa |
| *HaWRKY69* | Ha15_00037685 | Ha15 | 4253085 | 4258704 | 220 | 5.59 | 25.51 | plus | IIe |
| *HaWRKY70* | Ha15_00037730 | Ha15 | 5874901 | 5877081 | 324 | 8.85 | 36.69 | minus | IIc |
| *HaWRKY71* | Ha15_00037860 | Ha15 | 11935618 | 11937450 | 336 | 9.81 | 37.46 | minus | IId |
| *HaWRKY72* | Ha15_00037863 | Ha15 | 11991485 | 11993316 | 336 | 9.81 | 37.46 | minus | IId |
| *HaWRKY73* | Ha15_00038294 | Ha15 | 37030020 | 37034044 | 257 | 8.10 | 29.35 | minus | I |
| *HaWRKY74* | Ha15_00038388 | Ha15 | 44696095 | 44697936 | 321 | 8.55 | 35.37 | plus | IIa |
| *HaWRKY75* | Ha15_00038672 | Ha15 | 60313317 | 60314736 | 322 | 6.32 | 36.11 | minus | IIc |
| *HaWRKY76* | Ha15_00038745 | Ha15 | 66773103 | 66774290 | 307 | 5.62 | 35.13 | minus | IIe |
| *HaWRKY77* | Ha15_00039581 | Ha15 | 122654887 | 122658707 | 503 | 8.12 | 54.86 | minus | I |
| *HaWRKY78* | Ha15_00039958 | Ha15 | 150817754 | 150818636 | 148 | 9.17 | 17.46 | minus | IIIa |
| *HaWRKY79* | Ha15_00040149 | Ha15 | 165804616 | 165805792 | 305 | 9.63 | 33.22 | minus | IId |
| *HaWRKY80* | Ha15_00040607 | Ha15 | 196029196 | 196034033 | 290 | 9.81 | 31.63 | plus | IId |
| *HaWRKY81* | Ha16_00020764 | Ha16 | 7104158 | 7105532 | 285 | 8.68 | 31.48 | plus | IIa |
| *HaWRKY82* | Ha16_00021352 | Ha16 | 55014218 | 55016205 | 514 | 6.27 | 54.75 | minus | IIb |
| *HaWRKY83* | Ha16_00021557 | Ha16 | 75163971 | 75165004 | 296 | 9.98 | 31.95 | plus | IId |
| *HaWRKY84* | Ha16_00021908 | Ha16 | 99644584 | 99646888 | 523 | 5.86 | 58.06 | minus | IIb |
| *HaWRKY85* | Ha16_00021911 | Ha16 | 100098076 | 100100829 | 501 | 6.14 | 55.83 | minus | IIb |
| *HaWRKY86* | Ha16_00022468 | Ha16 | 143768973 | 143770838 | 257 | 4.81 | 29.04 | plus | IIe |
| *HaWRKY87* | Ha17_00008395 | Ha17 | 59343373 | 59345909 | 475 | 8.39 | 52.17 | plus | IIb |
| *HaWRKY88* | Ha17_00008399 | Ha17 | 59544692 | 59546621 | 262 | 5.95 | 30.47 | plus | IIa |
| *HaWRKY89* | Ha17_00008631 | Ha17 | 85962915 | 85966388 | 631 | 6.92 | 68.32 | minus | I |
| *HaWRKY90* | Ha17_00009615 | Ha17 | 188208187 | 188210261 | 229 | 8.43 | 25.97 | plus | IIIa |
