# Supplementary material for: Genome-wide characterization of WRKY gene family in Helianthus annuus L. and their expression profiles under biotic and abiotic stresses
Source: PLoS One. 2020 Dec 3;15(12):e0241965. doi: 10.1371/journal.pone.0241965 (PMC7714227; doi:10.1371/journal.pone.0241965)
Supplement: S3 Table — (DOCX) [file pone.0241965.s003.docx]

S3 Table. Corresponding gene names

| **In this study** | **Guo et al. 2019** | **Liu et al., 2020** |
| --- | --- | --- |
| *HaWRKY1* | *HaWRKY2* | *HaWRKY002* |
| *HaWRKY2* | *HaWRKY1* | *HaWRKY001* |
| *HaWRKY3* | *HaWRKY4* | *HaWRKY004* |
| *HaWRKY4* | *HaWRKY7* | *HaWRKY007* |
| *HaWRKY5* | *HaWRKY8* | *HaWRKY008* |
| *HaWRKY6* | *HaWRKY9* | *HaWRKY009* |
| *HaWRKY7* | *HaWRKY10* | *HaWRKY010* |
| *HaWRKY8* | *HaWRKY13* | *HaWRKY013* |
| *HaWRKY9* | *HaWRKY12* | *HaWRKY012* |
| *HaWRKY10* | *HaWRKY16* | *HaWRKY016* |
| *HaWRKY11* | *HaWRKY15* | *HaWRKY015* |
| *HaWRKY12* | *HaWRKY18* | *HaWRKY018* |
| *HaWRKY13* | *HaWRKY21* | *HaWRKY021* |
| *HaWRKY14* | *HaWRKY17* | *HaWRKY017* |
| *HaWRKY15* | *HaWRKY22* | *HaWRKY022* |
| *HaWRKY16* | *HaWRKY24* | *HaWRKY024* |
| *HaWRKY17* | *HaWRKY25* | *HaWRKY026* |
| *HaWRKY18* | *HaWRKY28* | *HaWRKY029* |
| *HaWRKY19* | *HaWRKY31* | *HaWRKY032* |
| *HaWRKY20* | *HaWRKY19* | *HaWRKY019* |
| *HaWRKY21* | *HaWRKY34* | *HaWRKY035* |
| *HaWRKY22* | *HaWRKY37* | *HaWRKY038* |
| *HaWRKY23* | *HaWRKY38* | *HaWRKY039* |
| *HaWRKY24* | *HaWRKY39* | *HaWRKY040* |
| *HaWRKY25* | *HaWRKY43* | *HaWRKY044* |
| *HaWRKY26* | *HaWRKY43* | *HaWRKY044* |
| *HaWRKY27* | *HaWRKY41* | *HaWRKY042* |
| *HaWRKY28* | *HaWRKY53* | *HaWRKY055* |
| *HaWRKY29* | *HaWRKY45* | *HaWRKY046* |
| *HaWRKY30* | *HaWRKY46* | *HaWRKY047* |
| *HaWRKY31* | *HaWRKY65* | *HaWRKY068* |
| *HaWRKY32* | *HaWRKY48* | *HaWRKY049* |
| *HaWRKY33* | *HaWRKY47* | *HaWRKY048* |
| *HaWRKY34* | *HaWRKY49* | *HaWRKY050* |
| *HaWRKY35* | *HaWRKY54* | *HaWRKY056* |
| *HaWRKY36* | *HaWRKY55* | *HaWRKY057* |
| *HaWRKY37* | *HaWRKY33* | *HaWRKY034* |
| *HaWRKY38* | *HaWRKY57* | *HaWRKY059* |
| *HaWRKY39* | *HaWRKY58* | *HaWRKY060* |
| *HaWRKY40* | *HaWRKY59* | *HaWRKY061* |
| *HaWRKY41* | *HaWRKY61* | *HaWRKY063* |
| *HaWRKY42* | *HaWRKY64* | *HaWRKY067* |
| *HaWRKY43* | *HaWRKY44* | *HaWRKY045* |
| *HaWRKY44* | *HaWRKY69* | *HaWRKY072* |
| *HaWRKY45* | *HaWRKY66* | *HaWRKY069* |
| *HaWRKY46* | *HaWRKY63* | *HaWRKY066* |
| *HaWRKY47* | *HaWRKY62* | *HaWRKY065* |
| *HaWRKY48* | *HaWRKY70* | *HaWRKY073* |
| *HaWRKY49* | *HaWRKY71* | *HaWRKY074* |
| *HaWRKY50* | *HaWRKY73* | *HaWRKY076* |
| *HaWRKY51* | *Not found* | *Not found* |
| *HaWRKY52* | *HaWRKY110* | *HaWRKY117* |
| *HaWRKY53* | *HaWRKY79* | *HaWRKY082* |
| *HaWRKY54* | *HaWRKY79* | *HaWRKY082* |
| *HaWRKY55* | *HaWRKY80* | *HaWRKY083* |
| *HaWRKY56* | *HaWRKY81* | *HaWRKY084* |
| *HaWRKY57* | *HaWRKY82* | *HaWRKY085* |
| *HaWRKY58* | *HaWRKY84* | *HaWRKY087* |
| *HaWRKY59* | *HaWRKY83* | *HaWRKY086* |
| *HaWRKY60* | *HaWRKY111* | *HaWRKY118* |
| *HaWRKY61* | *HaWRKY84* | *HaWRKY087* |
| *HaWRKY62* | *HaWRKY23* | *HaWRKY023* |
| *HaWRKY63* | *HaWRKY23* | *HaWRKY023* |
| *HaWRKY64* | *Not found* | *HaWRKY089* |
| *HaWRKY65* | *HaWRKY89* | *HaWRKY094* |
| *HaWRKY66* | *HaWRKY87* | *HaWRKY091* |
| *HaWRKY67* | *HaWRKY88* | *HaWRKY093* |
| *HaWRKY68* | *HaWRKY90* | *HaWRKY096* |
| *HaWRKY69* | *HaWRKY91* | *HaWRKY097* |
| *HaWRKY70* | *HaWRKY85* | *HaWRKY088* |
| *HaWRKY71* | *HaWRKY93* | *HaWRKY099* |
| *HaWRKY72* | *HaWRKY93* | *HaWRKY099* |
| *HaWRKY73* | *HaWRKY94* | *HaWRKY100* |
| *HaWRKY74* | *HaWRKY56* | *HaWRKY058* |
| *HaWRKY75* | *HaWRKY95* | *HaWRKY101* |
| *HaWRKY76* | *HaWRKY96* | *HaWRKY102* |
| *HaWRKY77* | *HaWRKY97* | *HaWRKY103* |
| *HaWRKY78* | *Not found* | *HaWRKY095* |
| *HaWRKY79* | *HaWRKY100* | *HaWRKY106* |
| *HaWRKY80* | *HaWRKY63* | *HaWRKY066* |
| *HaWRKY81* | *HaWRKY99* | *HaWRKY105* |
| *HaWRKY82* | *HaWRKY101* | *HaWRKY107* |
| *HaWRKY83* | *HaWRKY102* | *HaWRKY108* |
| *HaWRKY84* | *HaWRKY104* | *HaWRKY110* |
| *HaWRKY85* | *HaWRKY104* | *HaWRKY110* |
| *HaWRKY86* | *HaWRKY105* | *HaWRKY111* |
| *HaWRKY87* | *HaWRKY109* | *HaWRKY116* |
| *HaWRKY88* | *HaWRKY108* | *HaWRKY115* |
| *HaWRKY89* | *HaWRKY35* | *HaWRKY036* |
| *HaWRKY90* | *HaWRKY76* | *HaWRKY079* |
